# Neuroblastoma in neonates: a case report and literature review

**DOI:** 10.3389/fonc.2026.1776760

**Published:** 2026-03-17

**Authors:** Li Zhao, Guo Yu, Wang Jing, Kou Wei

**Affiliations:** West China Second University Hospital, Sichuan University, Sichuan, China

**Keywords:** MRI, neonates, neuroblastoma, parapharyngeal space, surgery

## Abstract

Neuroblastoma in neonates is a rare solid tumor. This report presents two rare cases of cervical neuroblastoma, both characterized by stridor and coughing during feeding due to tumor compression. The first case of neuroblastoma presented with an intact capsule, allowing for complete surgical resection with preservation of the cervical vasculature and nerves. In contrast, although preoperative MRI indicated an intact capsule in the second case, the intraoperative findings revealed no discernible capsule formation and involvement of both the internal and external carotid arteries. None of the two patients developed Horner’s syndrome postoperatively, and no evidence of recurrence or metastasis has been observed during the 10-month follow-up period. These cases also underscore that surgical intervention remains the primary treatment option for cervical neuroblastoma, particularly in alleviating the respiratory and feeding difficulties caused by tumor compression.

## Introduction

Neuroblastoma is a common pediatric solid tumor, with over 90% of cases occurring in children under the age of 5, predominantly around 2 years old, and rarely in newborns (1, 2). Tumors arise along the sympathetic nervous system, with the abdominal region being the most common site, followed by the thoracic region, while cervical involvement is the least frequent (3, 4). Neuroblastoma exhibits heterogeneous clinical presentations due to its propensity for metastasis and hormone secretion (5). Currently, tumor staging is based on the location of the tumor, the presence of distant metastasis, and the age of onset (6, 7). At the same time, risk group staging is conducted based on the tumor’s invasion of the blood vessels and organs and distant metastasis. In addition, previous studies on pediatric cervical neuroblastoma have demonstrated that surgical intervention, chemotherapy, and a variety of adjuvant therapies can lead to effective treatment outcomes. However, the pathological type and the *MYCN* gene status also affect the prognosis of patients (8).

## Case 1

**Figure 1:**
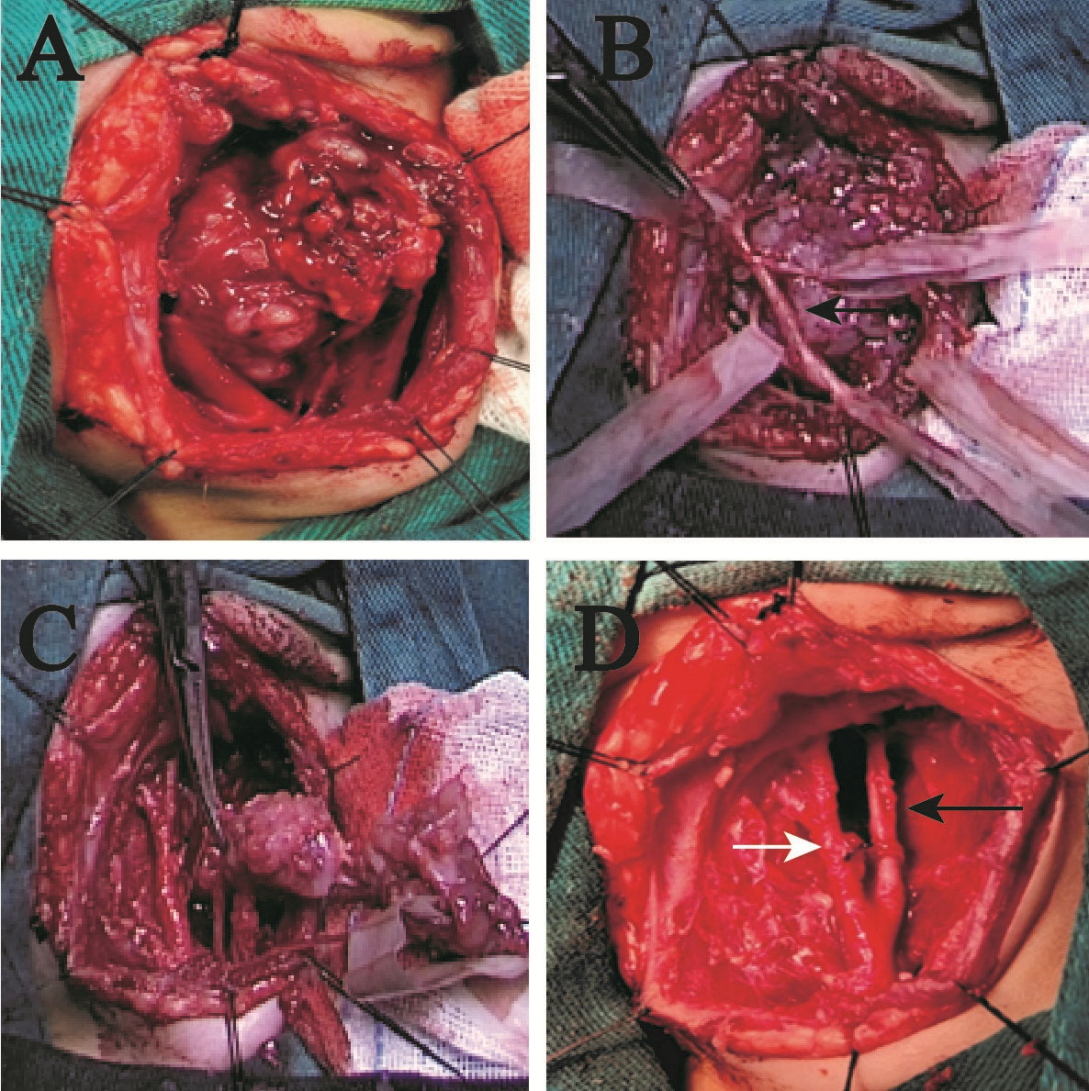
MRI of case 1. **(A)** T1 transverse section. **(B)** T2 transverse section. **(C)** T2 sagittal section. **(D)** T2 coronal section. The tumor measures approximately 33 mm in diameter and possesses an intact capsule. The black arrow indicates the internal carotid artery, while the white arrow denotes the external carotid artery, with occlusion observed in the internal jugular vein.

**Figure 2:**
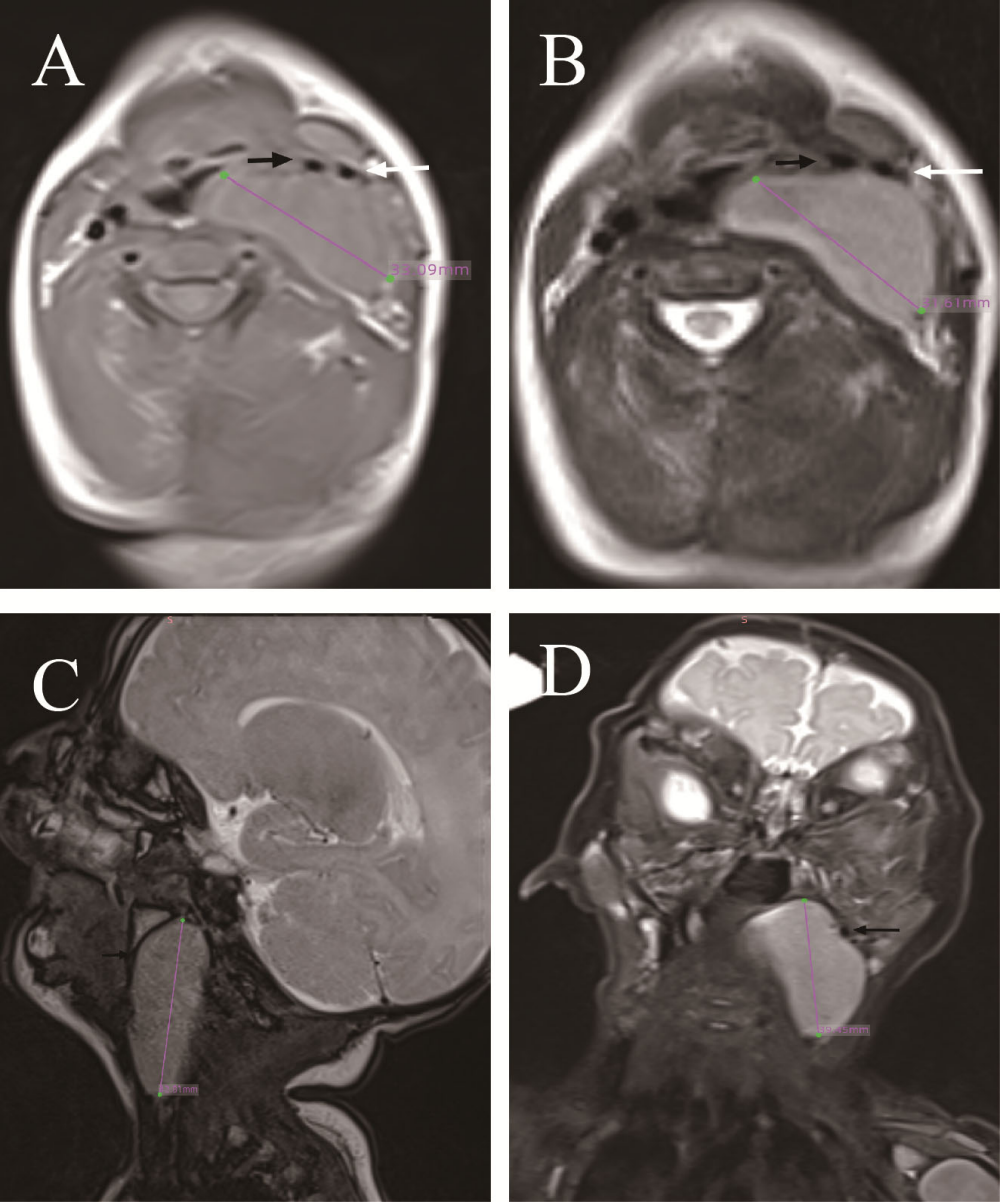
Pictures during the operation of case 1. **(A)** The carotid sheath vessels are pushed outward. Black arrow is the internal carotid artery (ICA), and white arrow is the external carotid artery (ECA). **(B, C)** The tumor has a complete mass, and the origin nerve was found above the tumor. The circle is the origin nerve. **(D)** The tumor was completely removed.

A male infant presented with a solid mass in the left cervical region identified shortly after birth, accompanied by inspiratory stridor and feeding difficulties with aspiration. There was no dyspnea, dysphagia, or hoarseness. With advancing age, the mass enlarged, accompanied by a gradual exacerbation of symptoms. Physical examination revealed a mass approximately 3.3 cm in diameter on the left side of the neck. No obvious lymph node enlargement was palpable on the opposite side or elsewhere. Laryngoscopy revealed compression of the left laryngeal cavity by the tumor, with normal movement and closure of both vocal cords. MRI examination revealed a solid mass in the left parapharyngeal space with an intact capsule, hypointense on T1 and non-uniform heterogeneous on T2, with no diffusion restriction. The tumor compressing the trachea and carotid artery is an International Neuroblastoma Risk Group (INRG) image-defined risk factor, which elevated the risk of surgery (**Figures 1A, B**). It extended into the pharyngeal cavity to the midline, pushing the internal and external carotid arteries laterally and compressing the internal jugular vein to the point of occlusion (**Figures 1C, D**). Intraoperatively, the tumor was found to be completely encapsulated (**Figure 2A**), the internal jugular vein was occluded, and the tumor’s nerve of origin was identifiable, exhibiting a terminal enlargement at the distal end of the nerve (**Figures 2B, C**). Postoperative inspiratory stridor and feeding-related aspiration were significantly improved, and no Horner’s syndrome was observed.

## Case 2

**Figure 3:**
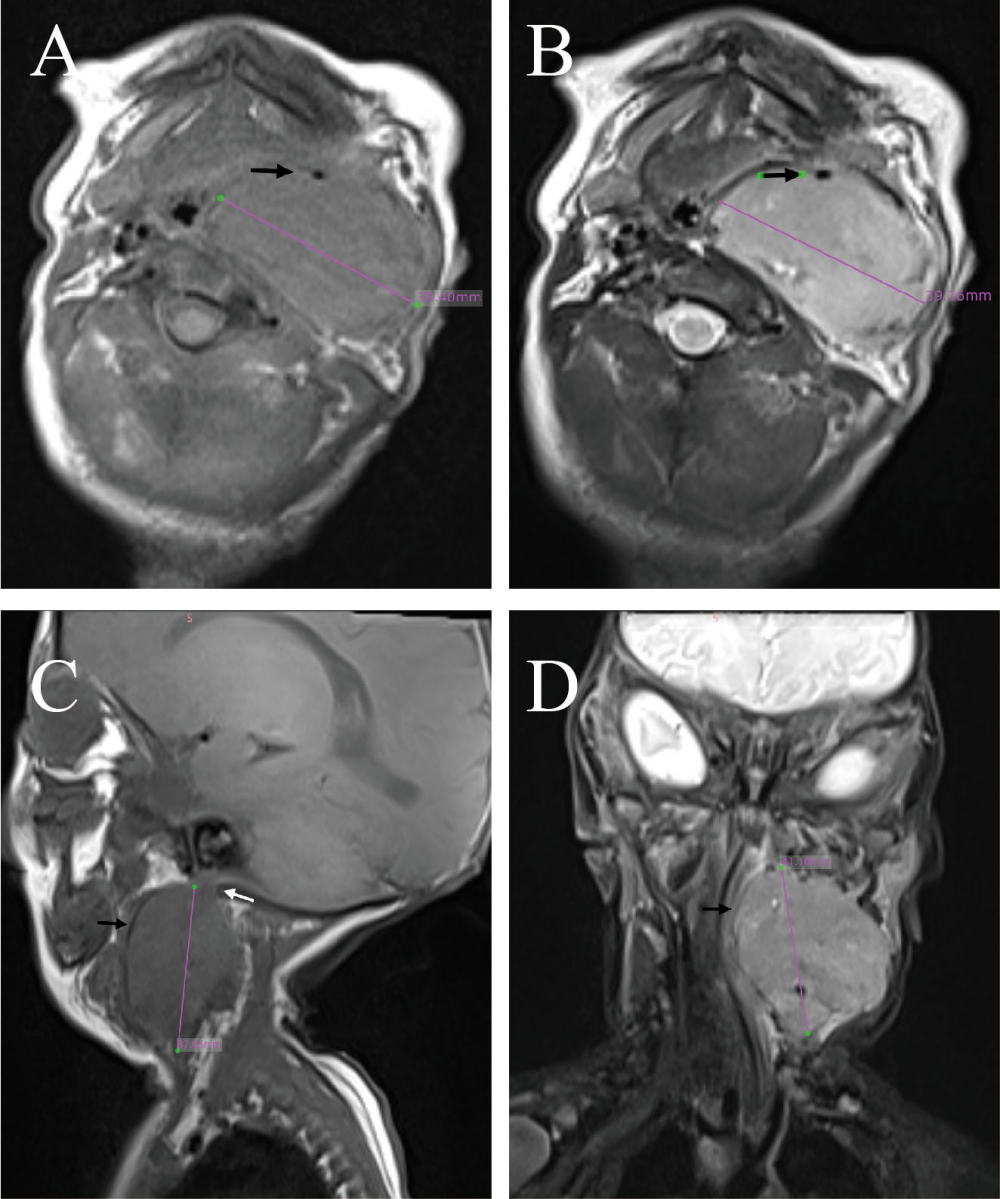
MRI of case 2. **(A)** T1 transverse section. **(B)** T2 transverse section. **(C)** T2 sagittal section. **(D)** T2 coronal section. **(A, B)** The tumor is encapsulated with a well-defined border. The black arrows indicate the internal carotid artery, with the external carotid artery and internal jugular vein showing occlusion. There is partial liquefactive necrosis in the center of the tumor. **(C)** Superior aspect of the tumor showing an indistinct boundary from the skull base tissues. **(D)** The tumor compressing into the laryngeal cavity.

**Figure 4:**
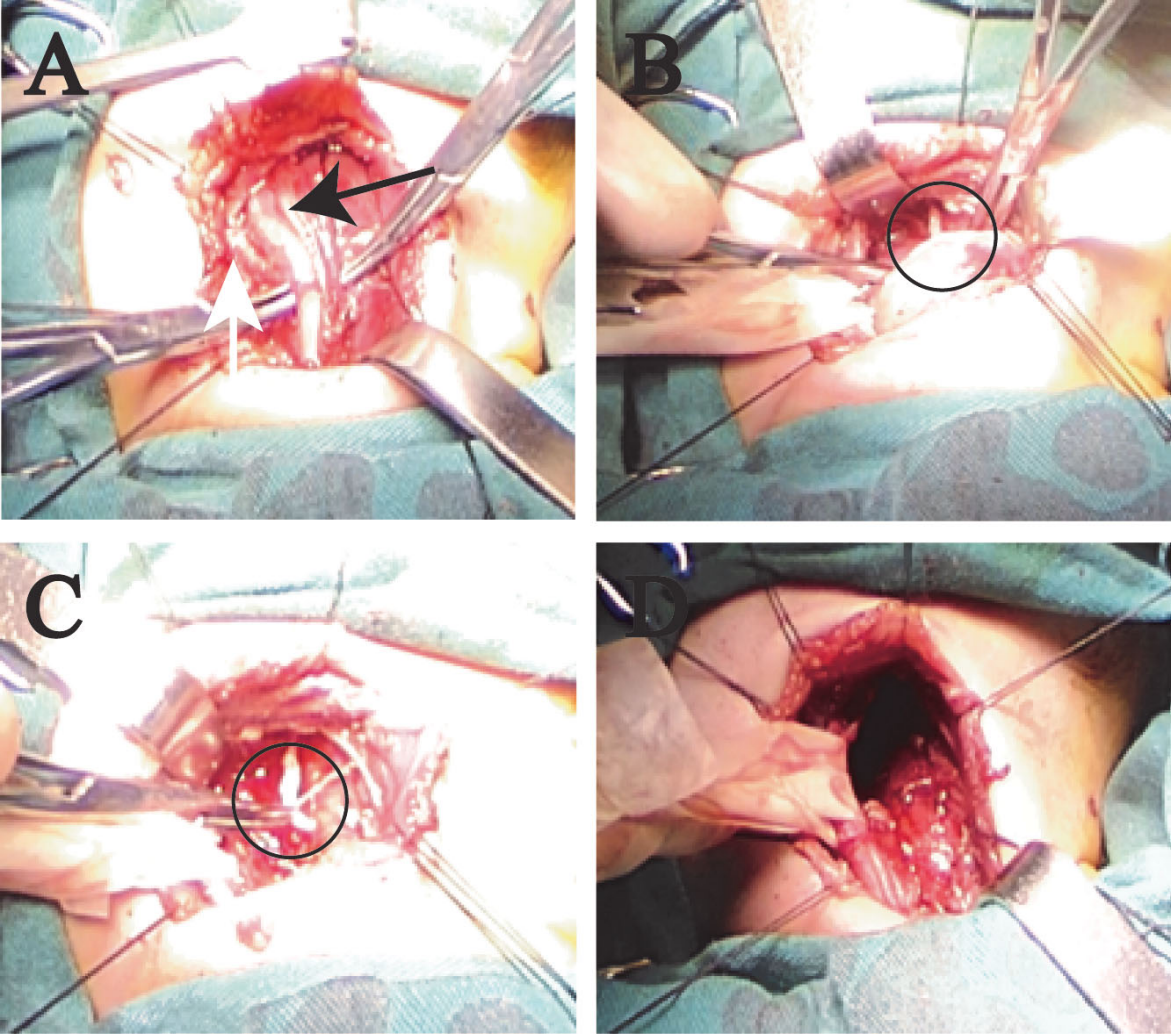
Pictures during the operation of case 2. **(A, B)** The tumor lacks a well-defined capsule and encases both the internal (ICA) and external carotid arteries (ECA). **(C)** The origin of the nerve was found at the lower end of the tumor. **(D)** The tumor was completely resected, necessitating sacrifice of the internal jugular vein. Black arrow is ICA and white arrow is ECA.

A male infant presented with a solid mass in the left cervical region identified shortly after birth, accompanied by hoarseness, inspiratory stridor, and feeding difficulties with aspiration. Dyspnea or dysphagia was absent. Physical examination revealed a mass approximately 4.2 cm in diameter on the left side of the neck. No obvious lymph node enlargement was palpable on the opposite side or elsewhere. Laryngoscopy revealed that the left vocal cord was fixed and that the glottis could not be completely closed. The MRI findings were essentially the same as those in case 1, but with partial liquefactive necrosis in the center of the tumor. The tumor compressing the trachea and surrounding the carotid artery is an INRG image-defined risk factor. Neither of the two patients had invasion of adjacent muscles or the base of the skull (**Figure 3**). Intraoperative examination revealed the tumor to be non-encapsulated, with partial encasement of both the internal and external carotid arteries (**Figures 4A, B**). The origin of the nerve was found at the lower end of the tumor, while no obvious nerve was found at the upper end (**Figure 4C**). Post-surgery, all symptoms resolved, with the exception of persistent hoarseness. No Horner’s syndrome was observed.

## Discussion

Neuroblastoma of the neck in neonates is extremely rare. Neuroblastoma of the neck originates from the sympathetic nerve in the neck (5). The most common initial symptom is a palpable mass or neck swelling, followed by breathing difficulties, fever, ear pain, and manifestations related to Horner’s syndrome (9). Neonates may present with characteristic symptoms such as laryngeal stridor and coughing during feeding due to compression by cervical masses. According to the INRG classification system, patients are stratified into the following treatment difficulty groups: very low risk, low risk, intermediate risk, and high risk. Current research findings indicate a strong association of *MYCN* gene amplification, 11q deletion, and chromosome 17q segmental alterations with high-risk disease profiles (4). The treatment outcomes for neuroblastoma exhibit a marked heterogeneity, with prognosis ranging widely from spontaneous regression to death (1). Surgery remains the main treatment for neuroblastoma, but is supplemented with chemotherapy based on the patient’s risk assessment and staging. The discordance between the MRI examination results and the findings during surgery indicates that the MRI results cannot be taken as the sole reference. There have been no reported cases on chemotherapy for neonatal cervical neuroblastoma; therefore, surgical intervention remains the primary treatment option for neonates presenting with significant clinical symptoms of this disease. In this article, the clinical symptoms of the two patients were significantly improved after surgical treatment. There were no related complications such as Horner’s syndrome. No recurrence was found 10 months after the operation. However, long-term follow-up is still required to monitor the patient’s subsequent clinical course.

There is currently no research indicating a direct correlation between the occurrence of neuroblastoma in children and maternal factors. Our follow-up investigations of the mothers of these two pediatric cases revealed no common gestational complications such as diabetes or hypertension, and the family medical history was negative for hereditary diseases.

## Conclusion

Neuroblastoma of the neck in neonates is a rare type of solid cervical tumor. Due to the presence of critical structures in the neck—such as the airway and arteries supplying blood to the brain—larger masses often lead to life-threatening clinical manifestations, including respiratory distress and compromised cerebral perfusion due to compression of the internal carotid artery. There are significant discrepancies between the MRI findings and the actual conditions during surgery in cases of neuroblastoma of the neck in newborns. Therefore, the MRI results should not be the sole reference before surgery. Prognosis is associated with multiple factors; however, surgical resection to relieve tumor compression remains the first-line therapeutic approach. Currently, there are no studies reporting on chemotherapy in neonates. Long-term follow-up is required to evaluate late recurrence and to determine the necessity of adjuvant chemotherapy. This study was conducted with the Ethics Committee and the parents of the child patient.

## Data Availability

The raw data supporting the conclusions of this article will be made available by the authors, without undue reservation.
